# Efficacy of Thoracic Duct Embolization in Managing Severe Refractory Chyle Leak Post-cervical Reconstruction in a Patient With Liver Cirrhosis: A Case Report

**DOI:** 10.7759/cureus.95040

**Published:** 2025-10-21

**Authors:** Masato Nagaoka, Hirokazu Ashida, Nobuki Fukuda, Katsuhiro Ishida, Hiromi Kojima

**Affiliations:** 1 Department of Otorhinolaryngology, Jikei University School of Medicine, Tokyo, JPN; 2 Department of Radiology, Jikei University School of Medicine, Tokyo, JPN; 3 Department of Plastic and Reconstructive Surgery, Jikei University School of Medicine, Tokyo, JPN

**Keywords:** chylous leak, conservative treatment, liver cirrhosis, neck dissection, thoracic duct embolization

## Abstract

This case report describes a patient with a history of liver cirrhosis who underwent neck reconstruction surgery and subsequently experienced substantial chyle leakage, reaching volumes of up to 6,620 mL per day. Conservative interventions such as fasting, a low-fat diet, total parenteral nutrition, drainage management, somatostatin analogs, and factor XIII supplementation proved ineffective, and the patient exhibited a refractory treatment course. The pronounced chyle leakage in this instance was hypothesized to be linked to increased lymph production resulting from portal hypertension associated with liver cirrhosis. Given the resistance to conservative treatment, thoracic duct embolization was performed by the interventional radiology department, effectively controlling the chyle leakage. The patient experienced a favorable clinical trajectory, devoid of complications. These findings indicate that thoracic duct embolization is a safe and efficacious minimally invasive treatment for refractory and massive chyle leakage following neck dissection and may represent a promising alternative before surgical intervention.

## Introduction

Because of the diverse anatomical pathways and delicate structure of the thoracic duct, it is susceptible to iatrogenic injury during head and neck surgeries that involve lower neck dissection. Accidental damage to the thoracic duct is relatively common and sometimes unavoidable, particularly during the resection of malignant tumors. Chyle, a lipid-rich lymphatic fluid, originates from the mesenteric lymphatics, traverses the cisterna chyli and thoracic duct, and ultimately enters the venous system at the left venous angle. In the context of head and neck surgery, injury to the cervical thoracic duct is identified as the primary cause of chyle leakage, which typically results in a greater volume of leakage compared with injuries to peripheral lymphatic vessels. Chyle leaks can lead to delayed wound healing, dehydration, nutritional disorders, electrolyte imbalance, and compromised immune function, making them a significant postoperative complication. Therefore, early diagnosis and appropriate therapeutic intervention for chyle leakage are essential to ensure a favorable postoperative outcome. Fundamental treatments for chyle leaks include conservative management such as fasting, a low-fat diet using medium-chain triglycerides, total parenteral nutrition, continuous drainage, and pharmacotherapy such as somatostatin analogs, etilefrine, and factor XIII supplementation [1-3]. In addition, when a lymphocele is detected, sclerotherapy to adhere to the cavity is commonly employed as a minimally invasive option [4]. However, for persistent chyle leaks that do not resolve with these methods, thoracic duct embolization or surgical procedures are viable treatment options [5,6]. Interventional radiology (IR) is a rapidly evolving field that has gained increasing attention in recent years because of its minimally invasive nature and therapeutic versatility. Here, we report a rare case of extensive postoperative chyle leak following cervical reconstruction in a patient with liver cirrhosis, which was successfully managed with thoracic duct embolization. This report highlights the clinical significance of thoracic duct embolization as a minimally invasive and effective alternative treatment for refractory postoperative chyle leakage.

## Case presentation

**Figure 1:**
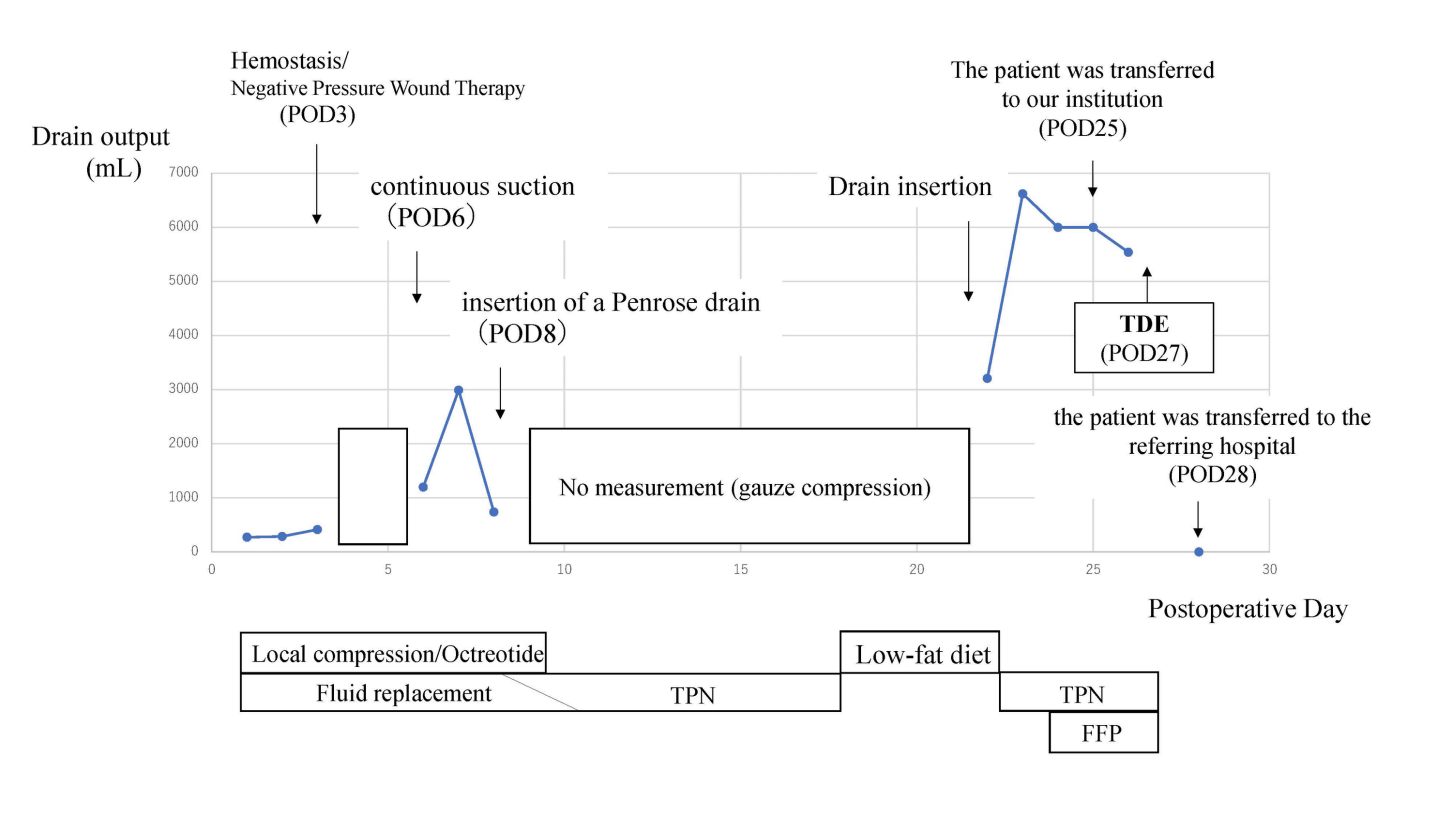
Postoperative course. In the early postoperative period, drain output slightly increased due to lymphatic leakage. However, following hematoma evacuation on postoperative day (POD) 3, the drainage markedly increased. Negative pressure wound therapy (NPWT) was initiated but proved ineffective, and subsequent placement of a continuous suction drain resulted in a further increase in output. Although conservative management temporarily reduced the volume, resumption of oral intake exacerbated the leakage, ultimately reaching a maximum of 6,620 mL/day. The patient was referred to our hospital, where thoracic duct embolization (TDE) was performed, leading to complete cessation of the chyle leak. He was transferred back to the referring hospital on the day after the procedure. FFP, fresh frozen plasma; TDE, thoracic duct embolization; TPN, total parenteral nutrition.

**Figure 2:**
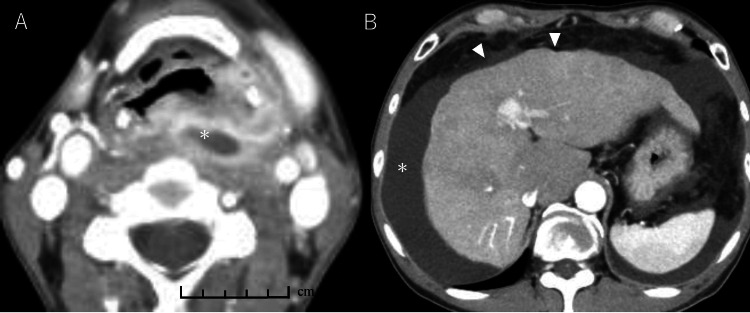
Contrast-enhanced CT of the neck and abdomen. (A) In the axial view of a contrast-enhanced computed tomography (CT) scan of the neck, a tumorous lesion (asterisk) is observed, primarily located in the left piriform recess. The lesion has partially extended beyond the thyrohyoid membrane, infiltrating the extralaryngeal space. (B) The contrast-enhanced CT scan of the abdomen reveals an irregular hepatic surface (arrowhead) and a substantial presence of ascites (asterisk).

**Figure 3:**
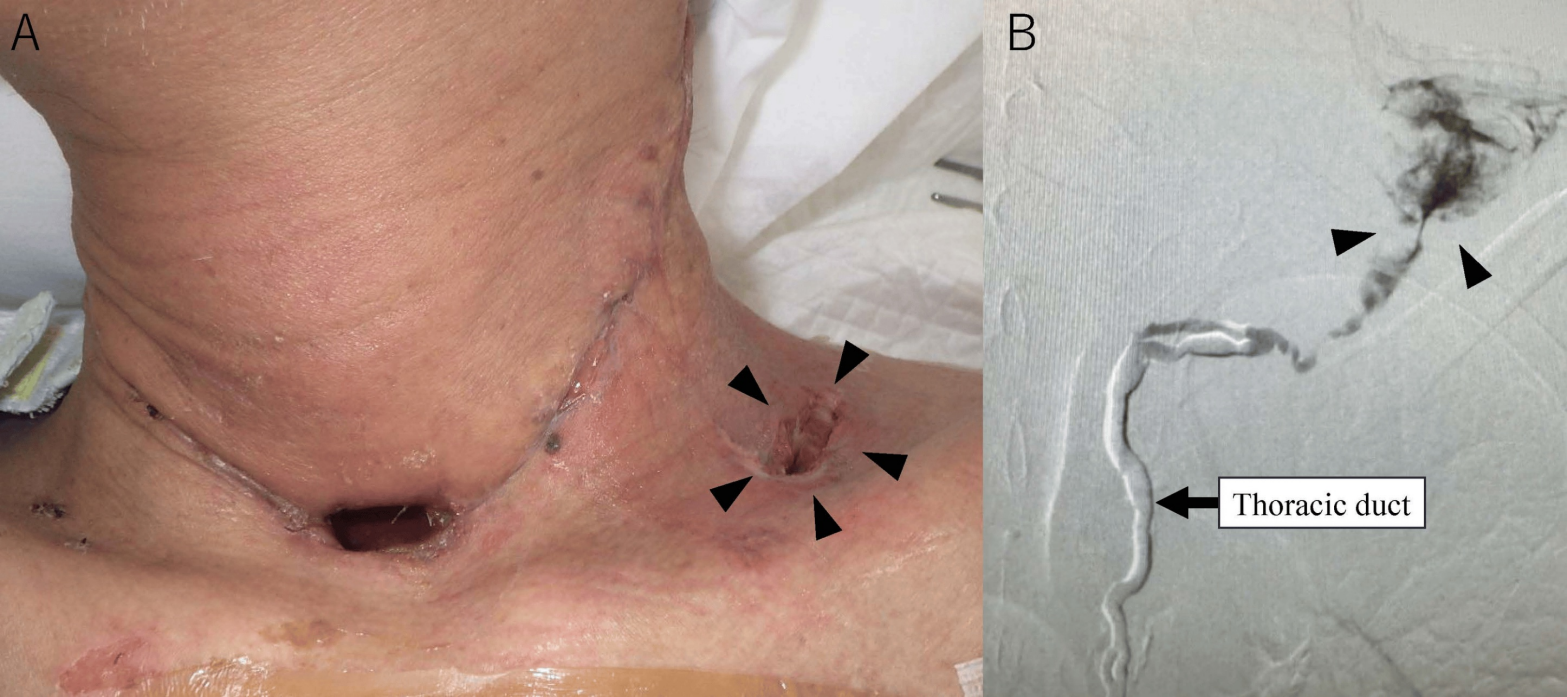
Imaging findings of postoperative fistula and chyle leakage. (A) The postoperative status following pharyngolaryngoesophagectomy and reconstructive surgery is demonstrated. A fistula is identified in the left supraclavicular region (arrowhead). (B) Lymphangiography of the thoracic duct revealed a disruption at the junction of the thoracic duct and left supraclavicular region, accompanied by chyle leakage (arrowhead).

**Figure 4:**
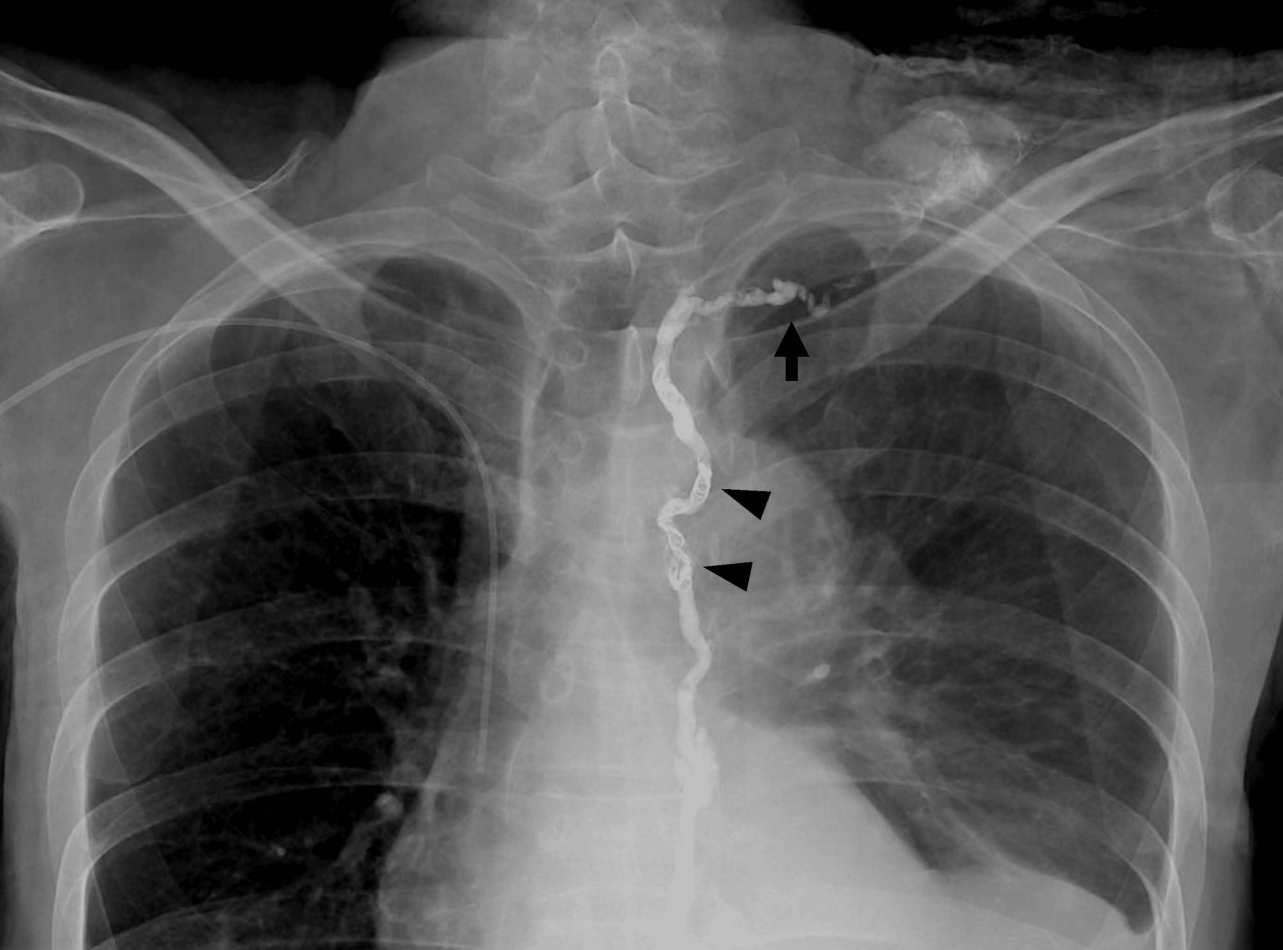
Thoracic duct embolization following lymphangiography. Coil embolization at the level of the aortic arch is distinctly visible (arrowhead). Residual contrast was noted in the peripheral segment of the thoracic duct; however, this was attributable to the retained contrast medium from the pre-embolization study (arrow).

A detailed summary of the patient’s clinical course is shown (Figure 1). The patient, a 56-year-old man, was diagnosed with hypopharyngeal cancer, classified as cT4aN2cM0 squamous cell carcinoma according to the latest AJCC/UICC 8th edition staging system (Figure 2A) [7]. His medical history included alcoholic liver cirrhosis (Child-Pugh class B, classified according to the Child-Pugh system) (Figure 2B) [8]. His medications included branched-chain amino acid granules (4.15 g, three times daily) and furosemide (20 mg, daily). In 2024, the patient underwent pharyngolaryngocervical esophagectomy, bilateral neck dissection, total thyroidectomy, and reconstruction with an anterolateral thigh musculocutaneous flap. Vascular anastomosis was performed using the right superior thyroid artery and common facial vein as recipients. On postoperative day 2, increased drainage from the left lower cervical drain suggested lymphatic leakage, which was treated with compression and octreotide. On day 3, hemostasis was achieved for oral bleeding, with a concurrent rise in blood pressure worsening the lymphatic leakage. The drain was removed, and negative pressure wound therapy (NPWT) was initiated. The negative pressure effect was lost shortly after initiation, necessitating the use of an electrically powered low-pressure continuous suction device. On postoperative day 6, continuous suction was initiated, but this resulted in a sudden and marked increase in lymphatic leakage. Therefore, on postoperative day 8, a Penrose drain was inserted, and cervical compression therapy was initiated. In addition, nutritional support was transitioned to total parenteral nutrition (TPN). A low-fat diet was initiated on day 18. However, on day 22, lymphatic leakage recurred, requiring drain placement, and the output reached 3,210 mL/day. By day 23, the drainage had increased to 6,620 mL/day. On day 25, he was referred to our hospital to undergo IR as a less invasive therapeutic option, given his history of liver cirrhosis. He received total parenteral nutrition (1,000 mL/day), acetated Ringer's solution (4,000 mL/day), and 6 units of fresh frozen plasma (FFP). On day 26, with a 5,540 mL/day output, he received similar nutrition with an increased amount of Ringer's solution (7,000 mL/day) and 12 FFP units. On the day following transfer, the drain was removed (Figure 3A). On day 27, lymphangiography and thoracic duct embolization (TDE) were performed. The inguinal lymph nodes were punctured with a 25G needle for lymphangiography using Lipiodol. The cisterna chyli was punctured with a 21G percutaneous transhepatic cholangiography (PTCD) needle, followed by microcatheter placement. After confirming extravasation (Figure 3B), the injured thoracic duct was embolized with coils and an NBCA-Lipiodol mixture (Figure 4). Drainage ceased on day 28, and the patient was transferred back to the previous hospital.

Serum sodium, potassium, and albumin levels remained within normal ranges during the perioperative period despite massive fluid and FFP replacement. Although albumin levels showed a slight decrease, this was consistent with the magnitude of surgical stress and did not represent a clinically significant deviation. In contrast, transient elevations in total bilirubin, PT, and PT-INR were observed. These changes are reasonably explained by the prolonged operative duration and perioperative physiological stress rather than pathological liver dysfunction (Table 1).

**Table 1:** Laboratory findings of the patient. Serum sodium, potassium, and albumin levels remained within the normal range throughout the perioperative course, despite massive fluid and FFP replacement. A slight decline in albumin was noted, which is not unexpected in the setting of an extensive surgical procedure, but no marked deviation was observed. Transient elevations in total bilirubin, PT, and PT-INR were observed, most likely attributable to the prolonged operative duration and perioperative physiological stress. FFP, fresh frozen plasma; PT, prothrombin time, INR, International Normalized Ratio.

Parameter	Patient value	Normal range
Total bilirubin (mg/dL)	0.4-4.1	0.2-1.2
Albumin (g/dL)	2.9-4.1	3.5-5.0
PT (%)	57.6-81.4	80-100
PT-INR	1.12-1.43	0.9-1.1
Sodium (mEq/L)	135-142	135-145
Potassium (mEq/L)	3.5-5.0	3.5-5.0

## Discussion

**Figure 5:**
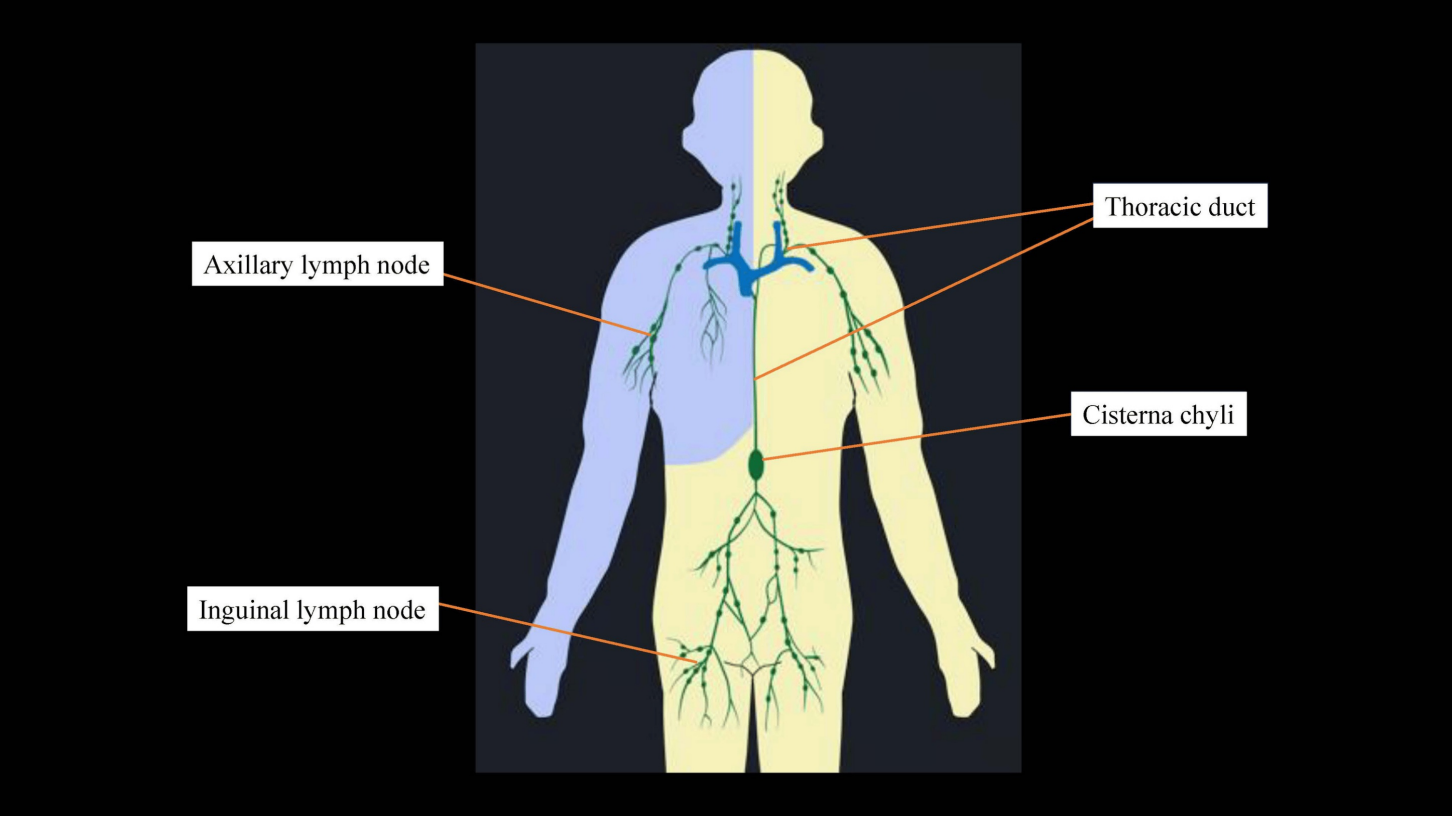
Lymphatic pathways of the whole body. Lymphatic drainage from the left upper limb and both lower limbs traverses the cisterna chyli and subsequently empties into the left venous angle via the thoracic duct, whereas lymphatic drainage from the right upper limb and the right side of the head and neck enters the right venous angle. This original illustration was created by the authors with reference to the educational 3D anatomy application “Visible Body.”

The thoracic duct carries chyle, a lipid-rich lymphatic fluid originating from the mesenteric lymphatics, through the cisterna chyli and into the left venous angle, where it typically drains into the left subclavian vein (Figure 5).

Consequently, left-sided neck dissection, particularly at level IV, carries an increased risk of thoracic duct injury. Postoperative chyle leaks occur in approximately 1-8% of head and neck cancer surgeries, with a particular tendency in procedures performed for thyroid cancer [2,9,10]. Although chyle leakage is an infrequent complication, its management can be challenging and may significantly affect patient recovery. According to the Clavien-Dindo classification system, high-output chyle leakage requiring interventional procedures, such as in the present case, corresponds to grade IIIb or higher, underscoring its clinical significance [11]. Prolonged lymphatic leakage may result in wound infection, malnutrition, immunosuppression, and severe systemic complications. Chylothorax may develop depending on the site of injury, and its mortality rate varies depending on the therapeutic strategy employed. Historically, mortality rates have reached as high as 25% in severe cases and those complicated by surgical interventions [12]. However, with advancements in less invasive surgical techniques and improvements in conservative treatment options, mortality rates have gradually declined.

In the present case, the pronounced chyle leakage was attributed to increased hepatic lymph production secondary to portal hypertension associated with cirrhosis, resulting in an augmented inflow into the thoracic duct [6,13]. Previous studies have proposed algorithms that classify chyle drainage based on volume into low-, high-, and very high-output categories [3]. Conservative management strategies for chyle leakage include fasting, a low-fat diet supplemented with medium-chain triglycerides, total parenteral nutrition, continuous drainage, and pharmacologic interventions such as somatostatin analogs, etilefrine, and factor XIII concentrate [1-3]. Although negative pressure wound therapy (NPWT) has been documented to facilitate the rapid closure of refractory chylous fistulas following neck dissection when conventional treatments have proven ineffective, it did not yield successful outcomes in the present case [14]. A previous case report indicated that balloon compression therapy administered between the internal jugular vein and sternocleidomastoid muscle was effective in a patient with a chyle leak of 1,100 mL/day after neck dissection. This underscores the potential of balloon compression as a minimally invasive therapeutic option for managing high-output chylous leakage [15]. Additionally, surgical management using a pectoralis major myocutaneous flap (PMMF) has been suggested as an effective method for controlling massive chyle leakage exceeding 4 L/day [6]. To the best of our knowledge, there have been no prior reports of a postoperative head and neck surgery case involving a chyle leak exceeding 6,000 mL/day that was successfully managed with nonsurgical treatment.

Direct interventions targeting the thoracic duct, such as IR procedures or surgical ligation, necessitate a thorough evaluation of their indications [16]. Among these interventions, thoracic duct embolization has gained attention as a minimally invasive technique with a high success rate and low complication rate [17]. The standard procedure involves diagnostic lymphangiography and percutaneous catheterization of the cisterna chyli or thoracic duct, followed by embolization of the leakage site. In cases of iatrogenic thoracic duct injury, lymphangiography has been reported to achieve an almost universal success rate, with catheterization success in approximately 67% of cases and an overall therapeutic success rate of 71%, including thoracic duct injuries. Notably, the complication rate was low, at approximately 3%, highlighting both the efficacy and safety of this procedure [5]. Although catheterization of the cisterna chyli may present technical challenges, lymphangiography itself has a high success rate and can be repeated if necessary, making it a robust therapeutic option to consider before surgical intervention. Similarly, recent reviews have supported the effectiveness of this approach in managing postoperative cervical chyle leaks [2]. Thoracic duct embolization or disruption may be performed depending on the drainage volume and extent of injury. In patients in whom catheterization is not feasible, thoracic duct disruption using a percutaneous transhepatic cholangiography (PTCD) needle has been demonstrated to be effective [5]. Furthermore, retrograde embolization of the thoracic duct via the venous system has been reported [18,19]. In the present case, thoracic duct embolization performed by the IR team was a minimally invasive and appropriate choice for a high-risk patient with a history of liver cirrhosis.

The patient exhibited persistent chyle leakage that was unresponsive to conservative management strategies, including fasting, a fat-restricted diet, high-calorie parenteral nutrition, compression, octreotide administration, and supplementation with FFP for factor XIII replacement. Although surgical thoracic duct ligation, including video-assisted thoracoscopic surgery (VATS), is considered a highly effective treatment option, perioperative risks remain a significant concern in high-risk patients with liver cirrhosis [20]. In this context, thoracic duct embolization performed by IR proved to be a highly effective and minimally invasive therapeutic approach for massive chyle leakage following head and neck cancer surgery in a patient with cirrhosis. These findings suggest that thoracic duct embolization is a valuable treatment option for refractory chyle leakage in high-risk patients.

## Conclusions

This case highlights the successful management of massive and refractory postoperative chyle leakage in a head and neck cancer patient with underlying liver cirrhosis. Conservative measures, including dietary modification, total parenteral nutrition, pharmacologic therapy, and supportive transfusions, proved ineffective. Thoracic duct embolization performed by the IR team resulted in rapid cessation of leakage and an uncomplicated recovery. These findings emphasize thoracic duct embolization as a safe and minimally invasive therapeutic alternative to surgical ligation, particularly in high-risk patients. This approach should be considered a valuable option in the treatment algorithm for refractory chyle leakage.
